# Is SUVmax Helpful in the Differential Diagnosis of Enlarged Mediastinal Lymph Nodes? A Pilot Study

**DOI:** 10.1155/2018/3417190

**Published:** 2018-10-28

**Authors:** Congcong Yu, Xiaotian Xia, Chunxia Qin, Xun Sun, Yongxue Zhang, Xiaoli Lan

**Affiliations:** ^1^Department of Nuclear Medicine, Union Hospital, Tongji Medical College, Huazhong University of Science and Technology, Wuhan 430022, China; ^2^Hubei Province Key Laboratory of Molecular Imaging, Wuhan 430022, China

## Abstract

**Objective:**

To explore the diagnostic value of maximum standard uptake value (SUVmax) from ^18^F-FDG PET/CT images in enlarged mediastinal lymph nodes of unknown etiology.

**Methods:**

We performed a retrospective study of patients with enlarged mediastinal lymph nodes on ^18^F-FDG PET/CT scans. SUVmax and the short axis and long axis of lymph nodes were recorded. These parameters were compared among the five commonest causes of mediastinal lymphadenopathy: lymphoma, metastatic disease, sarcoidosis, tuberculosis, and lymphadenitis. Histopathologic diagnosis was recorded as the final golden standard.

**Results:**

A total of 94 patients (62 men and 32 women; age range 7–85 y) were included with final diagnoses of 42 patients with benign pathology and 52 patients with malignancies. The sensitivity, specificity, and the accuracy of PET/CT in diagnosis of the benign and malignant mediastinal lymph nodes were 94.2%, 73.8%, and 85.1%, respectively. The SUVmax of benign and malignant groups were 13.10 ± 5.21 and 12.59 ± 5.50, respectively, which had no statistical difference (*P* > 0.05). However, the long axis and the short axis of lymph nodes in the benign and malignant groups were 2.86 ± 1.02 cm, 1.77 ± 0.60 cm and 6.04 ± 3.83 cm, 3.95 ± 2.08 cm, respectively (*P* < 0.05). The diagnostic values of PET/CT were higher than those of the long or short axis. However, the specificity of PET/CT was lower (73.8%) than that from the long or short axis (90.5% and 92.9%, respectively), although no statistical difference existed. Among the five common causes of mediastinal lymphadenopathy, significant differences could be seen in SUVmax and in the long axis and the short axis of lymph nodes (*P* < 0.05).

**Conclusions:**

SUVmax, a commonly used semiquantitative measurement, was not helpful for differentiation between benign and malignant lesions in patients with enlarged mediastinal lymph nodes in this study. Many benign lesions, such as sarcoidosis and tuberculosis, had high FDG uptake, possibly a trend that the size of the lymph nodes seems to have some diagnostic value.

## 1. Introduction

Unexplained mediastinal lymphadenopathy is not uncommon in clinical. Some patients visit a doctor due to dysphagia, hoarseness, or enlarged lymph nodes occasionally found in the physical examination. The symptoms may be caused by enlarged lymph nodes that compress the esophagus and recurrent laryngeal nerves. Lymph nodes may be enlarged due to benign or malignant etiologies. Early and accurate diagnosis and characterization of the etiology of mediastinal lymphadenopathy are essential to formulating a treatment plan.

The mediastinum is not an organ, but an anatomical area. In this area, there are several important tissues and organs, such as heart, large blood vessels, esophagus, trachea, thymus, nerves, and lymphatic tissue. Therefore, the mediastinal anatomy is complicated, and the tissue biopsy is difficult. There are invasive methods for evaluation of abnormal mediastinal lymph nodes, including mediastinoscopy (Med) [1], thoracoscopy [1], transbronchial needle aspiration (TBNA) [2], endobronchial ultrasound-guided transbronchial needle aspiration (EBUS-TBNA) [3], and endoscopic ultrasound-guided fine needle aspiration (EUS-FNA) [4]. The advantages of these methods are visual and intuitive and can be obtained with accurate pathological diagnosis. Some studies reported the sensitivity for Med, TBNA, EBUS-TBNA, and EUS-FNA in detecting malignancy were 80%, 78%, 89%, and 91%, respectively, and the specificity were 100%, 100%, 100%, and 100%, respectively [1–4]. The reason for the difference of the sensitivity may be related to the biopsy methods which could not access all the lymph nodes in mediastinum. For example, Med and EBUS-TBNA could not reach prevascular, subaortic, paraaortic, paraesophageal, and pulmonary ligament nodes [5]. Although these methods can obtain pathological results and have high specificity, they are invasive and may lead to complications. For example, TBNA can lead to mediastinal gas, bleeding, infection, and so on, while these incidence rates are low in EBUS-TBNA.

The traditional noninvasive examinations, chest computed tomography (CT) and magnetic resonance imaging (MRI), are the standard imaging modalities for assessment of mediastinal lymph nodes. However, MRI spatial resolution is relatively poor due to the presence of the air in the lungs, and the calcification of lymph nodes is often ignored by MRI [6]. CT could detect lesions, but it is also difficult to obtain the differential diagnosis of benign and malignant lymph nodes [6].

Positron emission tomography/computed tomography (PET/CT), integrating morphological imaging with functional imaging, is a noninvasive imaging method based on molecular functional imaging, which improves the diagnostic sensitivity and accuracy [7–9]. To some degree, PET/CT complements the deficiencies of traditional imaging and plays an important role in the workup of mediastinal lymphadenopathy. According to Nguyen's retrospective study, the sensitivity and specificity of PET/CT in the diagnosis of the benign and malignant mediastinal lymph nodes were 87% and 89%, respectively [10]. Also, the sensitivity, specificity, and the accuracy of PET/CT (87%, 91%, and 82%) in detecting mediastinal lymph nodes metastases were higher than CT (68%, 61%, and 63%) based on a recent report [11].

In the PET imaging analysis, standard uptake value (SUV), as a semiquantitative data, points off the degree of metabolic activity (aerobic glycolysis) in selected tissues [10]. The maximum standardized uptake value (SUVmax) is the maximum number of counts within the pixels in a region of interest (ROI). SUVmean is the mean number of counts in an ROI. SUVmax is preferred over SUVmean as there is a variability of about 35% between observers when SUVmean is used, and this reduces to 3% when SUVmax is used [12]. The SUVmax cutoff value of 2.5 is used commonly to differentiate between benign and malignant lesions [13]. Kumar et al.'s study of 35 cases of mediastinal lymphadenopathy showed that appropriately increasing the cutoff values can improve the specificity while maintaining an acceptable sensitivity [6]. When 2.5 or 6.2 was used as the cutoff value, the sensitivity, specificity, positive predictive value (PPV), negative predictive value (NPV), and accuracy were 93%, 40%, 54%, 89%, and 63% and 87%, 70%, 68%, 87%, and 77%, respectively [6]. There are a significant number of false positives (due to inflammatory diseases) and false negatives (due to low-grade malignancies) [14].

Research on unexplained enlarged mediastinal lymph nodes is relatively rare. This is mainly due to the complicated mediastinal anatomy, fewer pathology results, and number of cases, which makes the research impossible. Furthermore, since there are different views on the clinical value of PET/CT in evaluating enlarged mediastinal lymph nodes, it is difficult to draw consistent conclusions. In particular, the significance of SUVmax in diagnosing mediastinal lymph nodes has not yet been reported in detail. Hence, we planned to explore the clinical value of PET/CT images in enlarged mediastinal lymph nodes of unknown etiology, especially the diagnostic value of some quantitative and semiquantitative measures in the differentiation of malignant from benign lesions, such as SUVmax and lymph node size.

## 2. Subjects and Methods

### 2.1. Patient Population

This study was approved by the Institutional Review Board of Union Hospital, Tongji Medical College, Huazhong University of Science and Technology. Patients with enlarged mediastinal lymph nodes of unknown etiology and ^18^F-FDG PET/CT scans were included in this retrospective study. The following inclusion criteria were used to select patients: (1) the enlarged mediastinal lymph nodes were defined as the long axis >1 cm or generalized pulmonary hilar enlargement on CT images; (2) the enlarged mediastinal lymph nodes had higher FDG uptake than that of the adjacent blood pool; (3) the patients had not undergone treatment; (4) clinical data were complete, and formal follow-up was recorded; (5) histopathologic diagnosis was recorded as the final golden standard. Patients with diabetes were excluded.

### 2.2. Image Acquisition

All patients fasted for at least 6 hours before PET/CT examination. The images were obtained on a dedicated PET/CT scanner (Discovery VCT®, GE Medical Systems, Milwaukee WI, USA) 45–60 minutes after intravenous injection of 3.7–5.55 MBq/kg of ^18^F-FDG. A low-dose CT scan was obtained for attenuation correction, using the following parameters: tube voltage 120 kV, 80 mAs, and 3.75 mm slice collimation. PET images were acquired from the level of the head to the upper part of the legs (usually 6–8 bed positions) at 3 minutes per bed position. PET data were reconstructed with the ordered-subset expectation maximization algorithm. Both CT and PET data were sent to a workstation (Xeleris®, GE Medical Systems) for evaluation.

### 2.3. Image Analysis

Two experienced nuclear medicine physicians, who were familiar with the patient's clinical history, laboratory examinations, and traditional images (CT or MRI), independently reviewed all the PET/CT images and gave diagnosis separately. If the diagnosis disagreement happened, another two physicians participated in the discussion and finally reached an agreement about the final diagnosis from PET/CT images. An ROI was carefully drawn on the lymph nodes, and then the SUVmax was calculated according to the following formula:

(1) $$\begin{array}{c} \text{SUV}=\frac{\mathrm{Tissue}\;\;\mathrm{activity}\;\;\left(\text{MBq}/\text{mL}\;\;\mathrm{tissue}\right)}{\text{Injected}\;\;\mathrm{dose}\;\;\left(\text{MBq}\right)/\text{body}\;\;\mathrm{weight}\;\;\left(\text{g}\right)}. \end{array}$$

According to the new lung cancer lymph node distribution made by the International Association for the Study of Lung Cancer (IASLC), we located each lymph node and measured the long axis and short axis of the largest lymph node. If some lymph nodes were fused together, we measured it as one node [15].

### 2.4. Statistical Analysis

The data were collected and analyzed using commercial software (SPSS 19.0®, SPSS Inc., Chicago Il, USA). The SUVmax, the long axis, and the short axis of benign and malignant lymph nodes were compared using a two-sample *t*-test. A receiver operating characteristics (ROC) curve was drawn to find the best differential diagnostic point. The chi-squared test was used for multiple sample rates, and partitions of the *χ*^2^ method were used for multiple comparisons. The SUVmax, the long axis, and the short axis of lymph nodes among common mediastinal lymphadenopathy diseases were compared using the analysis of variance. These diseases included lymphoma, metastatic lymph nodes, sarcoidosis, tuberculosis, and lymphadenitis. Multiple comparisons between multiple samples were made using LSD (least significant difference), *t*-test (homogeneity of variance), and the Tamhane test (heterogeneity of variance). *P* values <0.05 were considered statistically significant. *P* values <0.0125 were considered statistically significant when using partitions of the *χ*^2^ method.

## 3. Results

There were 94 cases finally included in this study. Forty-two cases were found to have benign, and 52 had malignant etiologies on histopathology. Among the 42 benign pathologies, 16 were sarcoidosis, 17 were tuberculosis, eight were lymphadenitis, and one was Castleman disease. Among the 52 malignant pathologies, 25 were lymphoma, 26 were metastatic lymph nodes, and one was acute leukemic infiltration. The relevant features of all cases are summarized in Table 1.

### 3.1. Diagnostic Value of PET/CT, SUVmax, Long Axis, and Short Axis of Lymph Nodes in Benign and Malignant Lesions

**Figure 1:**
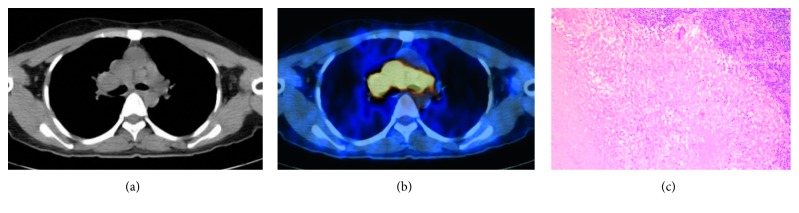
A 42-year-old female patient developed dry cough without fever. (a) Chest CT axial imaging showed enlarged lymph nodes in the mediastinum and right hilar areas. (b) PET/CT scan showed extensive hypermetabolic activity in the mediastinal and hilar lymph nodes (SUVmax 24.5). PET/CT indicated malignant lesions (lymphoma). The final pathological diagnosis was tuberculosis (c).

The sensitivity, specificity, PPV, NPV, and the accuracy of FDG PET/CT in diagnosis of the benign and malignant mediastinal lymph nodes were 94.2% (49/52), 73.8% (31/42), 81.7% (49/60), 91.2% (31/34), and 85.1% (80/94), respectively. Eleven false-positive PET/CT cases and three false-negative cases were found (Table 2). Lesions of tuberculosis were easily misdiagnosed as malignant lesions among these false-positive cases. In this study, eight of 17 patients with tuberculosis were misdiagnosed as malignant lesions, for a misdiagnosis rate of 47%. A typical case is shown in Figure 1 (case no. 69 in Table 2).

The SUVmax, long axis, and short axis of lymph nodes in the two groups are listed in Table 3. No statistical difference was seen in SUVmax between the malignant (12.59 ± 5.50, *n*=52) and benign cases (13.10 ± 5.21, *n*=42). The long axis and the short axis of lymph nodes in the benign and malignant groups were 2.86 ± 1.02 cm, 1.77 ± 0.60 cm and 6.04 ± 3.83 cm, 3.95 ± 2.08 cm, respectively (*P* < 0.05). These results indicated that SUVmax is not useful in determining whether the lymph nodes are benign or malignant; however, the size of the nodes measured on CT may provide more accurate information.

**Figure 2:**
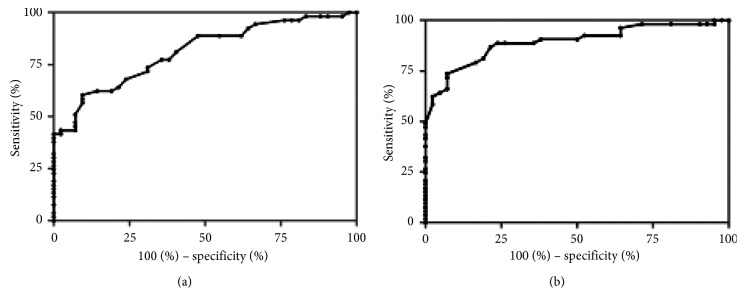
ROC curves of the long axis (a) and short axis (b) of lymph nodes in the differentiation between the benign and malignant diseases.

An ROC curve was drawn to find the best diagnostic differential point of the long axis and the short axis of lymph nodes in the distinction between benign and malignant diseases. The optimal threshold of the long axis of lymph nodes was calculated at 4.05 cm with 59.6% sensitivity, 90.5% specificity, 73.4% accuracy, and an area under the curve of 0.811 (95% confidence interval (CI) 0.726–0.896) (Figure 2(a)). The optimal threshold of the short axis of the lymph nodes was calculated at 2.55 cm with sensitivity 73.1%, specificity 92.9%, accuracy 81.9%, and an area under the curve 0.891 (95% CI 0.825–0.957) (Figure 2(b)).

The sensitivities of PET/CT, the long axis, or the short axis used separately to detect the benign and malignant mediastinal lymph nodes were statistically different in the chi-squared test, as well as the specificity (*P* < 0.05) (Table 4). When the sensitivities of the above three methods were compared separately by partitions of the *χ*^2^ method, the results were statistically significant for PET/CT and the long axis and PET/CT and the short axis (both *P* < 0.00125). These results indicated that the sensitivity of PET/CT was significantly higher than that of the long axis or the short axis used separately to detect the benign and malignant mediastinal lymph nodes. Although the specificity of PET/CT (73.8%) seems lower than that of the long axis (90.5%) or short axis (92.9%), the similar pairwise comparison of specificities showed no statistical significance (*P* > 0.0125). Taken together, the diagnostic efficacy of PET/CT was higher than that of the long axis or the short axis. Comparing the diagnostic efficiency of long and short axis, the short axis measurement was superior to the long axis measurement.

### 3.2. Diagnostic Value of PET/CT in Different Common Diseases of Mediastinal Lymphadenopathy

SUVmax, the long axis, and the short axis of five common causes of mediastinal lymphadenopathy are listed in Table 5. The three measures of five diseases were statistically different by the analysis of variance.

**Figure 3:**
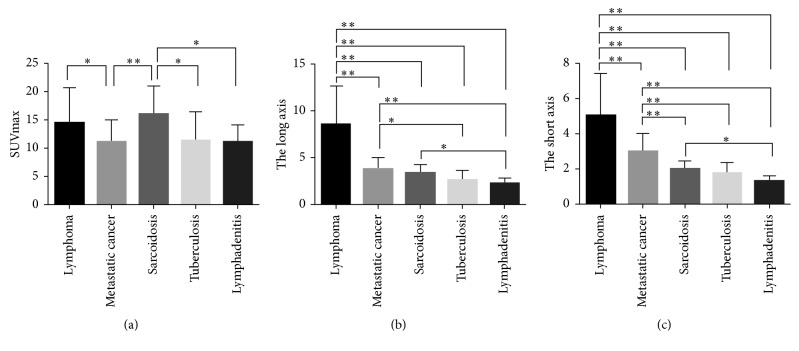
The comparison of SUVmax (a), long axis (b), and short axis (c) in five common mediastinal lymphadenopathy diseases (^*∗∗*^*P* < 0.01,^*∗*^*P* < 0.05).

Using the LSD-*t*-test, the pairwise comparison of SUVmax of five groups showed there are statistical differences. SUVmax of sarcoidosis is statistically higher than that of tuberculosis and lymphadenitis; however, it had no significant difference with that of lymphoma (Figure 3(a)).

Using the Tamhane test, the pairwise comparison of the long axis of five groups showed significant differences between lymphoma and all other diseases including metastatic lymph nodes, which indicated that the size of lymphomatous nodes was larger than that of the other lesions. The size of lymphadenitis nodes was smaller compared with the other diseases except tuberculosis (Figure 3(b)).

Using the Tamhane test, the pairwise comparison result of the short axis of five groups is shown in Figure 3(c). Obviously, the size of lymphoma and metastatic lymph nodes was significantly larger than that of benign lesions. These results indicate that the short axis of lymph nodes is important in the distinction between benign and malignant lesions.

## 4. Discussion

Enlarged mediastinal lymph nodes incidentally found on chest X-ray or CT need evaluation to determine their benign or malignant etiology. Because of the complicated anatomy of the mediastinum and the possible risk of tissue biopsy, noninvasive methods play an important role in the diagnosis of the benign and malignant mediastinal lymph nodes. In this study, a total of 94 patients with pathological diagnosis were included with 42 benign and 52 malignant etiologies on histopathology. The sensitivity, specificity, PPV, NPV, and accuracy of PET/CT in the diagnosis of the benign and malignant mediastinal lymph nodes were 94.2% (49/52), 73.8% (31/42), 81.7% (49/60), 91.2% (31/34), and 85.1% (80/94), respectively. This indicated PET/CT seemed to have some diagnostic value in mediastinal lymphadenopathy. However, SUVmax had no significant relationship with the benignity or malignancy of lesions in this set of cases. The long axis and the short axis of lymph nodes had a certain diagnostic value in benign and malignant lesions, with the risk of malignancy increasing with size.

PET/CT has been widely used for tumor diagnosis, differential diagnosis, staging, follow-up, therapy planning, and prognosis [16, 17]. In our cases, the accuracy of PET/CT in the diagnosis of the benign and malignant mediastinal lymph nodes was 85.1% combined with whole body PET/CT imaging and clinical information. Our results are consistent with prior research [6, 10].

There are a significant number of false-positive and false-negative PET/CT findings in the evaluation of primary tumors [14]. The major causes of false-positive lymph nodes are lymph node involvement by underlying inflammatory processes such as reaction to the presence of lung tumor, obstructive pneumonia, anthracosis, or granulomatous inflammation [18–21]. The major cause of false positivity may vary from region to region. In a study from Alabama, histoplasmosis infection was the most common cause of false positives [19]. Silicosis has been found to be a cause of false positives in a study from Germany [22]. In our study, patients with tuberculosis were easily misdiagnosed as malignant lesions among these false-positive cases, which accounted for 72.7% (8/11) of all misdiagnosed cases.

Mediastinal tuberculous lymphadenitis (MTL) is mostly seen in primary tuberculosis in children; it is uncommon in adults [23]. Absence of typical tuberculosis clinical features during the nonsuppurative lymphadenitis phase and age distribution characteristics makes the distinction between MTL and lymphoma and metastatic lymph nodes difficult, especially MTL during the active phase which has higher FDG uptake [23]. Patients with lymphoma usually have hyperpyrexia, hepatosplenomegaly, superficial chain lymphadenopathy, and obvious anemia. Homogeneous enhancement is more commonly seen in lymphoma than tuberculosis according to contrast-enhanced CT [24]. Metastases usually have a primary malignant disease. The commonest nodal metastases were from lung cancer, followed by gastroenteric tumor and prostatic cancer. For most metastases, diagnosis is not difficult after the primary disease has emerged [25]. Patients with sarcoidosis usually have chest, skin, and eye involvement. The CT scan of sarcoidosis usually shows symmetrical enlargement of bilateral hilar and peritracheal lymph nodes, which can be used to differentiate it from tuberculosis [24]. The enlarged lymph nodes mainly locate in the upper and middle zone of the mediastinum and more in the right side than the left side [24, 26]. In our study, the enlarged and fused lymph nodes of eight misdiagnosed cases had higher FDG uptake and a lack of typical tuberculosis clinical features with no caseous necrosis, which did not support tuberculosis. Hence, these findings need to be analyzed along with the clinical symptoms and laboratory test results.

The common causes of false negatives in the diagnosis of benign and malignant lesions are as follows. First, some low-grade tumors with lower FDG uptake may give rise to false negative results. Some researchers confirmed that the malignant tumor pathological type and degree of malignancy are closely related to FDG uptake [27]. There is a direct correlation between FDG uptake and extent of tumor invasion and growth rate. High-grade malignant tumors, bronchioloalveolar carcinoma, clear cell carcinoma, mucinous cell carcinoma, cystadenocarcinoma, and carcinoid often have low SUVmax measurements [28]. Second, smaller lymph nodes may give rise to false-negative results as well. The limited resolution of FDG-PET and the partial volume effect may prevent visualization of such small tumor deposits despite their potential accumulation of FDG [21, 29]. Several studies have shown a positive correlation between FDG uptake and the size of a lesion [30]. The threshold size of missed lesions is considered to be <8 mm [30]. A study from Takamochi showed that it was difficult for PET to detect metastatic lymph nodes measuring <5 mm [31]. In our study, three false-negative cases were misdiagnosed as benign lesions because the lymph nodes were all calcified and not fused, with pulmonary infection, and no evidence of a primary lesion or findings suggestive of malignancy on laboratory tests.

Our study found SUVmax was not of significant value in differentiating between benign and malignant mediastinal lymph nodes. The mean SUVmax in the benign group (13.10 ± 5.21) was greater than that in the malignant group (12.59 ± 5.50), which was different from the research of Kumar et al. With SUVmax of 6.2 as the cutoff as reported, the sensitivity, specificity, PPV, NPV, and accuracy were 87%, 70%, 68%, 87%, and 77%, respectively [6]. In our cases, the lymph nodes of the benign lesions had high FDG uptake, such as sarcoidosis (SUVmax 15.90 ± 5.07, *n*=16) and tuberculosis (SUVmax 11.29 ± 5.16, *n*=17). The further analysis of five common causes of mediastinal lymphadenopathy revealed that there was no significant difference between malignant lesions and sarcoidosis or tuberculosis.

SUVmax measured on PET/CT is a semiquantitative value that indicates the degree of aerobic glycolysis in a lesion [32]. In clinical diagnosis, the use of SUV in FDG-PET to diagnose cancer is an issue of ongoing controversy [10]. Interpretation of FDG PET is usually based on visual evaluation and not on SUV measurements because data have shown that the use of SUV failed to be more accurate than the visual evaluation in predicting the presence of malignancy [33, 34]. It is often assumed that FDG uptake is primarily within the malignant tumor cells and SUVmax is a well-known measure indicating the aggressiveness of the tumor [35, 36]. But other cellular components such as normal parenchymal cells, atypical cells, inflammatory cells, fibroblasts, or hematopoietic progenitor cells may also take up FDG [32]. The SUVmax cutoff value of 2.5 was used commonly to differentiate between benign and malignant lesions based on an early literature report [13]. Kumar et al.'s study of mediastinal lymphadenopathy showed that appropriately increasing the cutoff values can improve the specificity while maintaining an acceptable sensitivity [6]. When 5.3 or 6.2 was used as the cutoff value, the accuracy would be improved (74% or 77%) [6].

In this study, the long axis and the short axis of lymph nodes were helpful in distinguishing between benign and malignant mediastinal lymph nodes, especially the short axis. The bigger the lymph nodes were, the higher the possibility of malignancy was. The result was consistent with some other researchers' view of the short axis as the most accurate indicator in the diagnosis of malignant lesions [37, 38]. Among five common causes of mediastinal lymphadenopathy, the short axis of lymphoma and metastatic lymph nodes was larger than that of other benign lesions.

There is no accurate cutoff for the short axis of lymph nodes to differentiate benign from malignant lymph nodes. Using ROC curve analysis in our study, the optimal threshold of the short axis of lymph nodes was 2.55 cm with sensitivity 73.1%, specificity 92.9%, and accuracy 81.9%. The mean of the short axis in malignant groups (3.95 ± 2.08 cm) was greater than that in benign groups (1.77 ± 0.60 cm). The malignant lymph nodes are high-grade, fast-growing, and fuse, which leads to the increased size of malignant lymph nodes. But there is still a certain misdiagnosis rate for the following reasons: First, the response to the same disease varies from person to person, such as sluggish response in elderly, immature immune system in children, a strong response in young adults, different response between the strong person and the infirm person [39]. Second, early stages of the disease are easily misdiagnosed as benign lesions. Hence, the short axis of lymph nodes is still not very accurate in distinguishing benign from malignant.

In addition, digital pathology has the potential to transform the histopathological data more and more “real,” quantifiable and comparable to that of other disciplines such as nuclear medicine [40]. The examination of bioptic samples of patients subjected to PET/CT investigation can provide information about quantification of PET/CT targets or even the exact localization of the radiolabeled molecules in the tissues [40]. Taking advantage of this, a structured collaboration model between anatomic pathology and nuclear medicine can play a valuable role in the management of patients with unexplained mediastinal lymphadenopathy.

Our study showed that there was a certain value of PET/CT imaging combined with the size and metabolism of lymph nodes in the comprehensive evaluation of mediastinal lymphadenopathy. Although numerous studies have confirmed SUVmax has some value in the diagnosis of neoplastic diseases, SUVmax could not be the main index to distinguish between benign and malignant lesions, especially in locations where tuberculosis and other granulomatous disease are endemic. The integrated analysis of the PET/CT images and case history, clinical manifestation, laboratory tests, and a variety of imaging techniques is necessary. However, the size of the lymph nodes seems to have some diagnostic value, especially the short axis of lymph nodes.

Our study has some limitations. Firstly, since it is a retrospective study and has a limited number of cases, a study incorporating a large number of patients is needed. Secondly, because the enhanced CT was not performed, we were unable to accurately calculate the number of lymph nodes. The analysis based on lymph nodes would be much helpful. Thirdly, partial volume effect is not considered in this study, which is important to accurately correct the PET/CT signal in the lymph nodes. Moreover, metabolic tumor volume (MTV) and total lesion glycolysis (TLG) obtained from PET/CT images show more and more diagnosis and prognosis information. In the next work, we may continue to conduct the research about the role of MTV and TLG in the differential diagnosis of enlarged mediastinal lymph nodes.

## 5. Conclusions

SUVmax, a commonly used semiquantitative value for the lesion aerobic glycolytic rate, was not of significant value in patients with enlarged mediastinal lymph nodes in this study. Some benign lesions, such as sarcoidosis and tuberculosis, had high FDG uptake. Utilizing both the PET FDG uptake and CT characteristics including size and attenuation in an overall integrated report along with high quality clinical and laboratory data in a multidisciplinary meeting-like environment enables one more likely to reach the overall correct diagnosis for the patient.

## Figures and Tables

**Table 1:** Patient characteristic of 94 patients.

Variable	No.		
Age			
Range	7–85 y		
Median	50 y		
Sex			
Male	62		
Female	32		
Follow-up time (d)			
Range	43–1100		
Median	462		

Pathologic diagnosis	No. patients	Age (y) (range/median)	Male/female

Benign pathology	42	18–85/52	21/21
Sarcoidosis	16	28–57/50	6/10
Tuberculosis	17	19–75/50	10/7
Lymphadenitis	8	18–85/65	4/4
CD	1	53	1/0
Malignant pathology	52	7–78/47	41/11
Lymphoma	25	7–78/34	21/4
Metastatic lymph nodes	26	23–71/58	20/6
AL	1	64	0/1

CD: Castleman disease; AL: acute leukemic.

**Table 2:** False positive and negative cases diagnosed by ^18^F-FDG PET/CT.

Case no.	Sex	Age	SUVmax	Long axis (cm)	Short axis (cm)	PET/CT diagnosis	Pathological diagnosis
*False-negative cases*							
31	F	44	9.1	2.9	2.3	TB	Adenocarcinoma (high grade)
36	M	53	7.7	1.9	1.5	Lymphadenitis	Adenocarcinoma
93	F	64	10.6	3.1	1.9	Lymphadenitis	Leukemia infiltration
*False-positive cases*							
69	F	42	24.5	2.7	2.5	Malignant disease	TB
70	M	71	3.9	2.3	1.4	Malignant disease	TB
71	M	19	14.8	2.6	1.6	Lymphoma	TB
73	F	52	9.9	1.5	1.3	Malignant disease	TB
81	M	61	6.9	4.0	2.3	Malignant disease	TB
82	M	50	9.2	5.0	1.3	Malignant disease	TB
83	F	32	15.5	2.6	1.6	Lymphoma	TB
84	M	25	12.9	3.8	3.3	Malignant disease	TB
86	F	66	16.7	2.9	1.8	Malignant disease	Lymphadenitis
92	M	53	16.7	4.4	3.0	Malignant disease	CD
94	F	57	11.6	1.9	1.3	Malignant disease	Lymphadenitis

F: female; M: male; TB: tuberculosis; CD: Castleman disease.

**Table 3:** Comparison of the SUVmax and size of the lymph nodes in the benign and malignant lesions.

	Benign (*n*=42)	Malignant (*n*=52)	*t*	*P*
SUVmax	13.10 ± 5.21	12.59 ± 5.50	0.458	0.648
Long axis (cm)	2.86 ± 1.02	6.04 ± 3.83	−5.238	<0.001
Short axis (cm)	1.77 ± 0.60	3.95 ± 2.08	−6.573	<0.001

**Table 4:** Comparison of PET/CT, long axis, and short axis diagnostic efficacy.

Methods	Sensitivity (%)	Specificity (%)	Accuracy (%)
PET/CT	94.2	73.8	85.1
Long axis	59.6	90.5	73.4
Short axis	73.1	92.9	81.9
*χ* ^2^	17.186	7.389	4.323
*P*	<0.001	0.025	0.115

**Table 5:** The comparison of SUVmax, long axis, and short axis of lymph nodes in the common causes of mediastinal lymphadenopathy.

Diseases	*n*	SUVmax	Long axis (cm)	Short axis (cm)
Lymphoma	25	14.36 ± 6.35	8.51 ± 4.13	5.03 ± 2.40
Metastatic lymph nodes	26	10.97 ± 4.11	3.77 ± 1.29	3.00 ± 1.00
Sarcoidosis	16	15.90 ± 5.07	3.34 ± 0.91	1.98 ± 0.47
Tuberculosis	17	11.29 ± 5.16	2.60 ± 1.04	1.74 ± 0.63
Lymphadenitis	8	10.90 ± 3.16	2.25 ± 0.59	1.28 ± 0.32
*F*	—	3.529	23.594	21.386
*P*	—	0.010	<0.001	<0.001

## Data Availability

The data used to support the findings of this study are available from the corresponding author upon request.
